# Brain-immune interactions in health and disease

**DOI:** 10.3389/fnins.2014.00382

**Published:** 2014-12-03

**Authors:** Adam Denes, Jaleel A. Miyan

**Affiliations:** ^1^Faculty of Life Sciences, University of ManchesterManchester, UK; ^2^Laboratory of Molecular Neuroendocrinology, Institute of Experimental MedicineBudapest, Hungary

**Keywords:** brain-immune interactions, neuroinflammation, systemic responses, bi-directional communication, disease

Modern medicine cannot avoid the understanding of the fine-tuned communication between the many, seemingly distinct systems in the body. An excellent example of this is the challenge to understand the bi-directional communication between the brain and the immune system. Brain-immune interactions take place in different organs, involving a wide range of cells and mediators, coordinated through sensory and effector pathways in the central nervous system (Ader et al., 1990; Elenkov et al., 2000; Rivest, 2009). The interactions work in both directions to maintain a healthy state of body and brain in the face of diverse, harmful challenges from foodstuffs, toxins, allergens, infective agents, or injury. Dysfunction and inappropriate regulation of inflammatory or neuronal responses underlie many diseases that have become more prevalent in recent decades, predominantly in developed countries. These countries are also characterized by an increased aging population and profoundly increased cost to healthcare due to age-related brain conditions including dementia and other neurodegenerative diseases. Recent research has established a significant role for the immune system in several brain diseases including multiple sclerosis, tumors, stroke, mental disorders, Alzeimer's, and Parkinson's disease. In turn, mood disorders, stress, autonomic dysfunction, acute, and chronic brain injury have been linked with the development of organ failure, cancer, heart disease, systemic inflammatory conditions, infections, and hematological diseases further implicating dependent interrelationships between the immune system and the brain (Denes et al., 2010; Moreno-Smith et al., 2010; Deretzi et al., 2011; Iadecola and Anrather, 2011; Wraith and Nicholson, 2012; Theoharides et al., 2013; Heneka et al., 2014). Both preclinical and clinical research have contributed significantly to our knowledge about these interactions, yet another major challenge is to translate multiple research findings into clinical benefit.

The papers in this research topic discuss some of the most pressing issues concerning the interactions between the neural and immune systems. Murakami and colleagues present their research findings and their “gateway” theory of how regional neuronal responses can drive the migration of autoreactive T cells across the cerebrovascular endothelium to particular sites of the brain where they contribute to the development of experimental autoimmune encephalomyelitis (EAE), a mouse model of multiple sclerosis (Kamimura et al., 2013). They also show that regional neural stimulation can therapeutically prevent the gating through blood vessels. Geenen et al. discuss how autoimmunity directed against neuroendocrine glands could be due to genetic or acquired problems that affect the presentation of neuroendocrine self-peptides in the thymus (Geenen et al., 2013). This process in the thymus is normally responsible for the clonal deletion of self-reactive T cells and the generation of regulatory T cells. Their findings could thus support the development of novel treatment strategies against type 1 diabetes for example.

In their comprehensive review article, Anrather and colleagues describe how reprogramming of local and systemic immune mechanisms contributes to the induction of cerebral ischemic tolerance, a process that is characterized by protection against the ischemic injury after application of ischemic stress to one tissue or organ (Garcia-Bonilla et al., 2014). Appropriate reprogramming of key immune mechanisms could be used to develop novel stroke therapies including possible prevention of injury through stroke in vulnerable individuals. The research paper by Denes et al. demonstrates that brain injury, anesthesia, and surgical interventions have diverse systemic consequences, including altered leukocyte responses in several organs of the body and rapid mobilization of granulocytes (Denes et al., 2013). This could have important implications for animal models of cerebral ischemia as well as for patients with brain injury or for those undergoing surgeries or exposed to prolonged anesthesia. The review article by Möller and his colleagues focuses on the regulation of the kynurenine pathway by inflammatory mediators and how this contributes to neurodegenerative and psychiatric disorders (Campbell et al., 2014). They also highlight the potential for therapeutic interventions by modulation of the kynurenine pathway.

Assas and colleagues discuss important aspects of neuro-immune communication and show how sensory fibers containing the neuropeptide calcitonin gene-related peptide (CGRP) shape the responses of macrophages, mast cells and other immune cells throughout the body and how these interactions contribute to immune defense and diverse inflammatory conditions (Assas et al., 2014). This neuropeptide and the c class nerve fibers that contain it thus form a key pathway for bi-directional neuroimmune interactions and could form a target for future neuroimmune based therapies.

Neuro-immune abnormalities not only affect adults and the elderly, but also play a role in diverse diseases that manifest in children. D'Angiulli et al. show that children in the Mexico City Metropolitan Area, who are chronically exposed to high concentrations of air pollutants, present with increased amounts of inflammatory mediators along with accumulation of misfolded proteins in the cerebrospinal fluid (Calderon-Garciduenas et al., 2013). They propose that environmental factors could mediate detrimental actions in the developing brain.

Paul Ashwood and colleagues report that the behavioral characteristics, including social deficits, repetitive grooming behavior and atypical vocalizations, observed in BTBR T+tf/J mice are associated with the development of an inflammatory macrophage phenotype in this strain (Onore et al., 2013). They suggest that such a relationship between elevated inflammatory burden and repetitive grooming behavior may have relevance to the repetitive and stereotyped behavior characteristic of autism since many Autistic children also present with an increased inflammatory profile. Goyal and Miyan review the possible role of neuro-immune abnormalities in autism (Goyal and Miyan, 2014). They highlight the influence of environmental factors on the abnormal neurological, immunological, and neuroimmunological functions reported in Autistic children and discuss how these interactions can lead to or exacerbate autism spectrum disorder. Their discussion links poor development of the neuroimmune system to vulnerability to these environmental challenges and the consequential effects on the brain and its functions.

We hope that the articles presented in this research topic give thought-provoking and valuable insight into some of the important aspects of brain-immune interactions. Neuro-immune processes are likely to contribute to diverse pathologies in both the periphery and the brain leading to complex human diseases that affect millions of people worldwide. Understanding mechanisms of neuro-immune interactions could help to find appropriate therapies to some of these conditions.

## Conflict of interest statement

The authors declare that the research was conducted in the absence of any commercial or financial relationships that could be construed as a potential conflict of interest.
